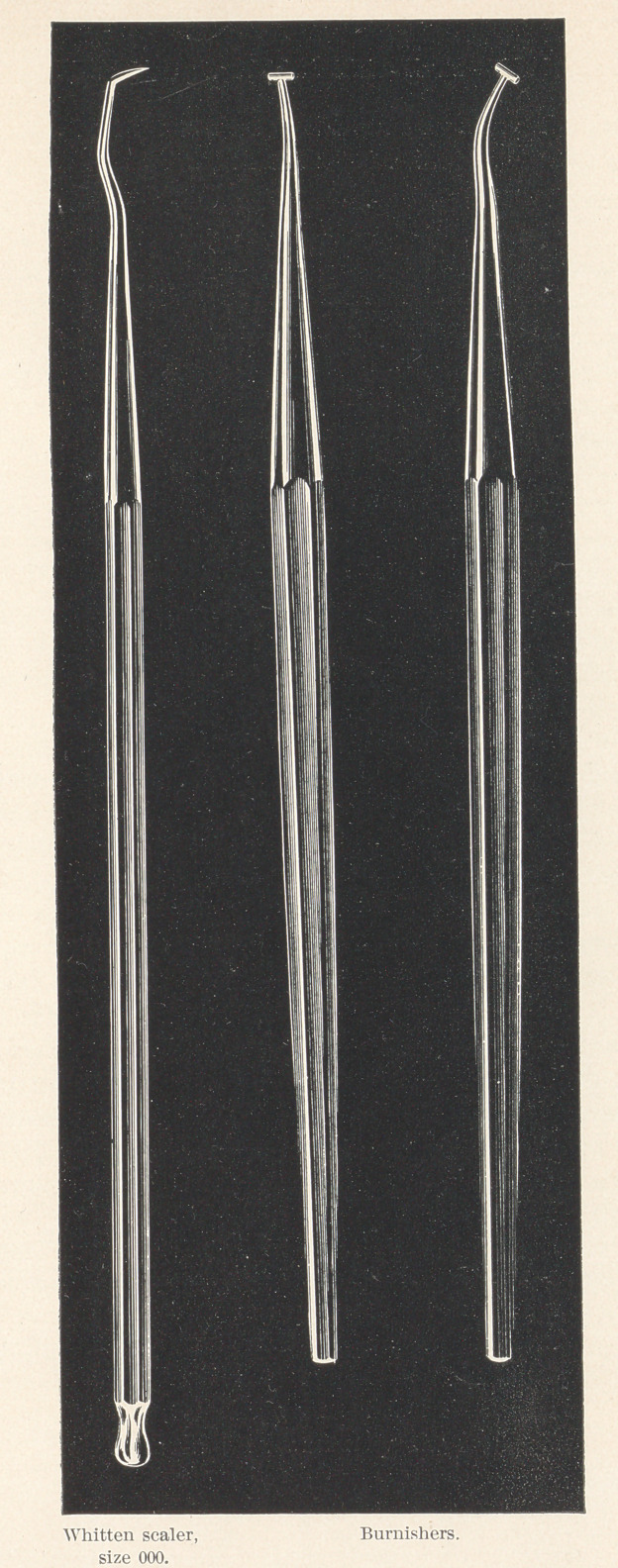# Some Uses for English Tube-Teeth

**Published:** 1903-01

**Authors:** Horatio C. Meriam

**Affiliations:** Salem, Mass.


### International Dental Journal.

VOL. XXIV. JANUARY, 1903. No. 1.

### Original Communications.[1]

### SOME USES FOR ENGLISH TUBE-TEETH.[2]

BY HORATIO C. MERIAM, D.M.D., SALEM, MASS.

IT would be a pleasant task if one could recall the various forms and methods used in crowning and give the merits of each their fitting place, but to-night I am to confine myself to some uses for English tube-teeth. I shall have the pleasure of first showing some of the ways that they may be used as crowns; then as they may be fastened to a cap or ring around an adjoining tooth, limiting myself to the bicuspid teeth as far as possible. Then refer to some of the instruments and methods of working, and present some general considerations bearing on the teaching of methods. Those who wish for a fuller account than can be given to-night I refer to an article by Mr. John Girdwood, L.D.S. (Edin.), D.D.S. (Univ. of Pa.), read before the World's Columbian Dental Congress and printed in Vol. I. of its transactions.

Of the two mechanical means now used in attaching crowns, the dowel, commonly called a pivot, was the method used until the gold

---

[1] The editor and publishers are not responsible for the views of authors of papers published in this department, nor for any claim to novelty, or otherwise, that may be made by them. No papers will be received for this department that have appeared in any other journal published in the country.

[2] Read before the American Academy of Dental Science, April 2, 1902.

cap came into use. This may be said to have given us the mortise and tenon, the cap representing the mortise and the root to be crowned the tenon; and we combine both when we use a pin or dowel in connection with a banded crown. The details of root preparation may be passed over, but later the importance of being guided by their anatomy will be pointed out. We have in the English tube-teeth a fine piece of porcelain with a hole through it lined with a platinum tube, which guarantees uniformity of diameter. This porcelain may be ground and polished without injury to its surface, a fine texture fits it admirably for matching certain colors in the natural teeth, and the hole through it allows for all the forms of attachment that can possibly be made by dowelling or its equivalent (Fig. 1, *a*).

We have, after grinding the tooth to fit the root and seeing that it has the right occlusion, the simple form of a dowel passing through the tooth into the root (Fig. 1, *b*).

Next we have the tooth concaved on its surface against the root to hold cement or gutta-percha as a preservation (Fig. 1, *c*); to this may be added the grinding out to allow the use of a headed dowel or pin (Fig. 1, *d*).

Some years since Mr. Hodge brought some hard Butler points made by vitrifying. I then knew little of the process, but when I became familiar with the processes of the Waltham Wheel Company I had some made there. Since the Indian oil-stone has been introduced I have had some made of that material which are suitable for this countersinking, as are also some forms made of carborundum and rubber, though these may not keep their shape as well as the harder material. Pins may be made by fusing to the end, if platinum, a globule of pure gold (Fig. 2, *a*), or if of spring gold wire (Fig. 2, *b*), which is often used, by fusing the end into a globule, and then passing it through a hole in a suitable anvil or a draw-plate, driving it down flat; or a small piece of plate may be soldered to the end of the pin (Fig. 2, *c*), the pin afterwards being held in a chuck and the head filed to fit the countersunk hole in the grinding surface of the crown. A long screw may be used, though screwing a screw up the centre of a root is not to be recommended, nor has the use of a headed pin much to commend it. One of the advantages of the use of these teeth is that we can insert the pin, see that the gutta-percha is packed well about it, and then slip the

FIG. 1.

FIG. 2.

Fig. 3.

Fig. 4.

Fig. 5.

crown on. The pin may, of course, be tapped and a small screw put in or a tube used, should it be desirable to crown a tooth before the drainage of pus has ceased in cases under treatment. This may often be desirable in the incisors, and for this these teeth are specially adapted (Fig. 2, *d*). I am taking it for granted that you are all familiar with the platinum and iridium tubes that were introduced in the Academy some years ago. My own choice is a pin with a slot cut in the end as you would for a screw, and then, after this has been placed in the root and the tooth put on, filling this and the countersunk space with gold-foil (Fig. 3, *a*).

This covers some of the ways in which a dowel may be used. We now take those cases where it is judged best to use a banded crown. For this band a firmer gold should be made than is ordinarily used for crowns, for it often has to be made narrow, and in some cases to bear much strain, so that the gold and solder the formula for which was presented to the Academy not long since should be used. The band should be first fitted to the root and then placed on the round part of an anvil, and the upper edge that is to receive the crown spread by striking with a small round-faced hammer; then annealed and placed root end down on a piece of lead or air-chamber metal, and the tooth, previously ground to correspond with the root diameter of the band, protected by another piece of the soft metal and driven in. It may be necessary to remove and reanneal to spread the ring a little more. To show what can be done, a molar tooth is reproduced here (Fig. 5). It was made some years since to show students. Such a stretching would not take place in soft gold, as the ring would go down under the hammer. The bands may be made narrow or not as the case indicates, and cut away on the buccal surface, this being reinforced if need be. In cases where the bite is short it is best to let the band come well down, so as to meet the occlusion of the under teeth on the palatal surface, that the strain may be taken off of the porcelain; this surface can also be reinforced (Fig. 4, *a*).

Teeth may also be ground so as to be much inclined inward or outward to meet occlusion or to harmonize with those adjoining. One such case is shown here (Fig. 3, *b*). Where it is desired to cap the root without letting the band run on to the crown the cap may be made as usual (Fig. 3, *c*, *d*), with the centre pin running through, and the tooth put on and finished in any of the ways previously shown. In some short bites it is an advantage to have the pin take the force of occlusion and protect the porcelain.

All that has been shown should be understood to refer to the second bicuspids or to teeth with one straight root and canal. It is desirable to make a distinct break before passing to the methods for treating the first superior bicuspid. To the solemn prayer of the Litany for the multiplied sorrows of life may well be added a petition,—that the Lord would indue the hearts and minds of his servants in our calling with mercy for the first superior bicuspid. The violence done to its anatomy by the diskers and contourists in its earlier years is followed by the reamer of the fitter of the ready-made crown, and its sad fate may well justify a special petition in its behalf. It is no doubt a credit to tooth-makers that we use anything that they choose to make without murmuring, but is it to us? Recall for a moment a section of an ordinary first bicuspid made near the gum line (Fig. 4, *b*), then a longitudinal section of the same root (Fig. 4, *c*), and name the tooth that you can buy ready made in the shops that is fitted to be used in crowning it. Its anatomy should dictate absolutely what method and crown should be used.

I reproduce here a cut of a pin shown by Mr. John Girdwood in his paper before alluded to, who states that it was devised by his partner, Mr. John Stewart, L.D.S. It has been in use by me for many years, and as I have not reported it I can advise it the more heartily as the credit for its introduction must be given to another. Mr. Girdwood shows it as made in two ways by soldering an upright to the forked cross-pin (Fig. 6, *a*), and by soldering a pin to the side (Fig. 6, *b*). I have never used the latter method. Mr. Girdwood seems to use but one size of wire. My practice has been to use No. 17, English standard gauge, for the tooth, and a smaller size for the root, varying the latter to correspond with the size of the root-canal. In small teeth the ends of the bifid pin may be made small enough to take the curve of the canal. They have also been made by using a wire one-half the size of the hole in the tube-tooth, twisting this for the length of the crown (Fig. 6, *c*), dividing and fitting to the root, and flowing a little gold solder over the twisted portion. The root of this tooth is often wide, in its palatal-buccal diameter, and there is sometimes a little trouble in having the crown portion of the pin in the right position; to overcome

Fig. 6.

Fig. 7.

Fig. 8.

this I use a half-round wire of the same number; the forked part, too, may be fitted first, then the wire bent around it, and compressed but not fastened (Fig. 6, *d*). The pin may then be tried in, the crown slipped on, and, when the right position has been found, removed and soldered. This pin may prove of value when there is great variation between the hole in the tooth and the root-canal, by using a pin bent at a right angle and the half-round wire bent and soldered to it. All that has been given regarding cap and band applies to this tooth, but the cap should have a pin for each root and a centre pin for the crown, to be fastened, preferably, by a gold filling (Fig. 7, *a*).

A method of using tube-teeth for extension crowns was given before the Academy some years since, and need only be recalled here, but the cup and pin shown (Fig. 7, *b*) can be used when it is desired to insert a bicuspid by banding or capping the first molar. The cup is made by fitting the tooth to the band as given earlier in the paper, a round cup with a pin in it is burnished over the upper portion of the tooth and band, care being taken to bend the edges of the cup over so as to indicate the relative position of cup and band, and the tooth is then removed and the cup and band soldered together. This cup is placed against the cap (Fig. 7, *c*) on the molar and soldered to it.

If a band is used on the molar it should be reinforced to give sufficient strength when cut away on the buccal side, and it should come to a sharp occlusion with the under teeth at its distal surface. It is best to wedge well and by reinforcing at this point secure contour. The distal occlusion helps to hold the band in position. In one case a large cavity has been filled with amalgam after putting on the band, and in another the band has been cut and fitted to correspond with the cusps of the molars. The treatment of another case is here shown where the band was extended backward to secure occlusion with a superior third molar (Fig. 8). Several cups could be used by soldering together for small bridges, removable if desired.

I have purposely left some things for mention after giving general directions. The importance of contour for crowns should not be overlooked, both as a protection to the membranes around the teeth and to aid in keeping the root in position. The bicuspid teeth move outward if they are given only a partial occlusion. They do not call for sharp cusps, but the occlusion should be even to keep them in line, and they should be large enough at the neck to cover the root, and extend beyond far enough to equal the protection once given by the natural crown. I would call attention to the Whitten scaler for trimming. I was with the late Dr. Whitten when he gave directions to Mr. Schmidt for making them, and I reproduce the 000 size here, because, while it is well known in Boston, it is not in other cities, and because for some of the work in trimming roots it is the best instrument that I know. This leads to the control of bleeding while trimming roots. For this, tincture of iodine is preferred, for it penetrates deeply and contracts the vessels when some agents only coagulate the blood at the surface. The cheek may be protected during grinding by using a cotton-fibre pad, which adheres to the cheek for a time and allows for easy holding away from the wheel. Small squares of bibulous paper, for root drying, folded diagonally and rolled, are of value. The squares are made by sending a thousand or more sheets to a book-binder and having him cut them to the desired size. It is much better than cotton for wrapping around a broach, as it goes on evenly. In the use of a broach it is well to dip it into peroxide. It can be neutralized by bicarbonate of soda if there is fear of its acting on the steel of the broach.

Polishing is dirty work. Ready cleansing of the hands can be secured by making a polishing composition containing soap,—one part powdered Castile soap and two parts of whatever polishing substance is to be used, to which add a small quantity of oil and a little water, all to be heated together and poured or pressed into a small paper box. This can be used in polishing, and the hands readily cleansed for operating. In polishing, large buffing wheels six or eight inches in diameter give the polish such as we see on jewelry. Felt wheels of the same size give smoother work than smaller ones. Cutter, Wood & Stevens Company, 68-70 Pearl Street, Boston, supply these shown. Fine chalk, called by dealers in painters' supplies French white, is smoother than whiting.

You may have noticed the lines on the pins shown. These are made by the checkering or grooving file introduced by Dr. Benjamin Lord for lining scalers. A wire is here shown to indicate how easily it can be serrated, as the file cuts only lines. A pin is easily forged cold from platinum and iridium, and then lined or serrated. These files can be had from F. W. Gesswein Company, 39 John Street, New York, whose catalogue contains much of value. It is there that many of the dental dealers get

Whitten scaler, size 000.

Burnishers.

their supplies. I show some burnishers which are of value in burnishing bands or gold crowns against the root on the buccal and palatal surfaces. A piece of rattan notched on the end to fit a crown is an aid in driving to place.

I fear that I have wandered far and taken you a dreary way in following the subject, but a call of the Academy cannot be lightly disregarded. If something of more value may be brought out in discussion I shall be well pleased. I hoped, by showing what could be done with one form of tooth, to broaden the subject, so that we could see the part it can have in our calling. Especially is this true of teaching; no one device or crown will do for all. Nor can the man taught only ready-made methods serve his profession or patient best. Professor McVeagh spoke here of the teaching of history at Harvard as not teaching what writers of history said, but taking men back to the study of original documents,—that is, to the source. Professor Dicey, of Oxford, said of the teaching of law at Harvard that "they were teaching the law of the English-speaking people." Many of us can recall the teaching of Dr. Bowditch in physiology, that it was the study of how nature acted in man, and how little of it we could read up. And if this is true for law, history, and medicine at Harvard, why not for our calling? Hard examinations are of little value in themselves; the result of the training should be that in addition to competent workmen it gives us men with active intelligence in matters relating to dentistry. One favorite study will liberalize the mind, and by making our art a liberal art we shall broaden as our study broadens, and daily duties will become daily pleasures.

I said a moment ago I had wandered far, but not perhaps farther than the subject has led me. Art is long, let us include as much as we can; and go back for a moment to the great prophet of Israel: "They helped every one his neighbor, and every one said unto his brother, be of good courage. So the carpenter encouraged the goldsmith, and he that smootheth with the hammer him that smote the anvil, saying, It is ready for the soldering: and he fastened it with nails, that it should not be moved."

## Figures and Tables

**Fig. 1. f1:**
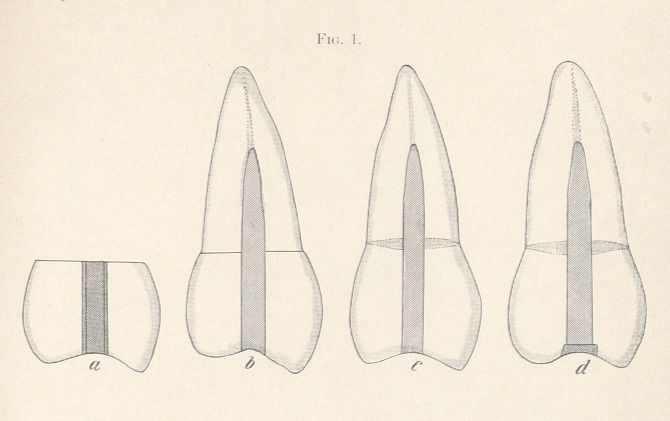


**Fig. 2. f2:**
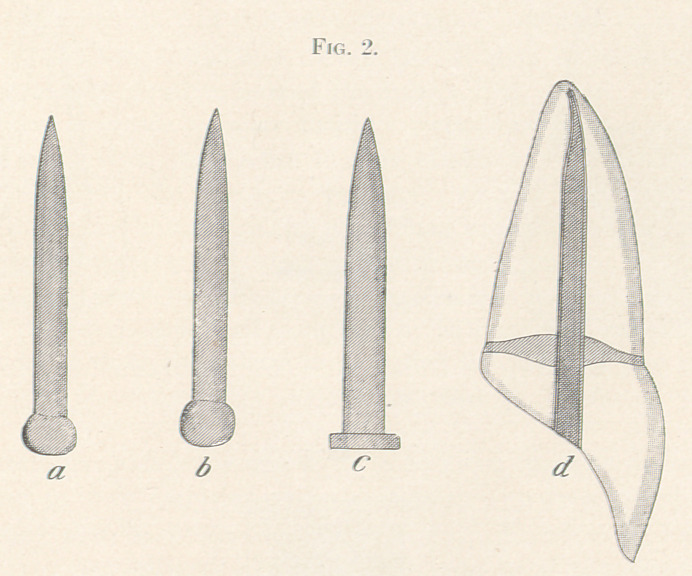


**Fig. 3. f3:**
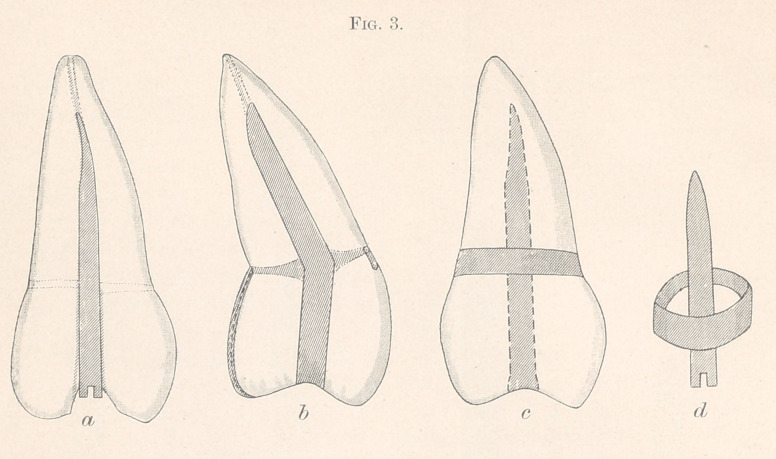


**Fig. 4. f4:**
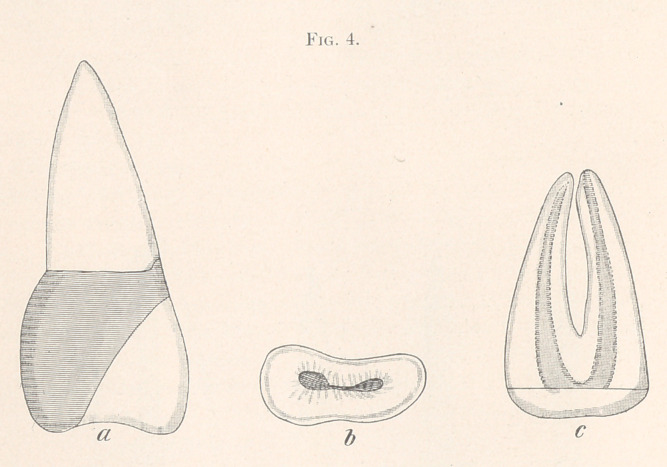


**Fig. 5. f5:**
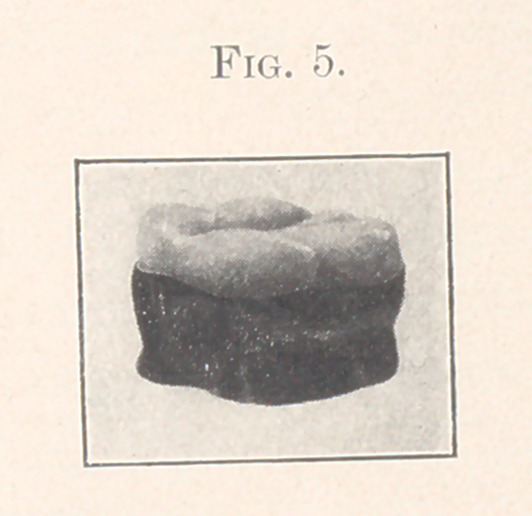


**Fig. 6. f6:**
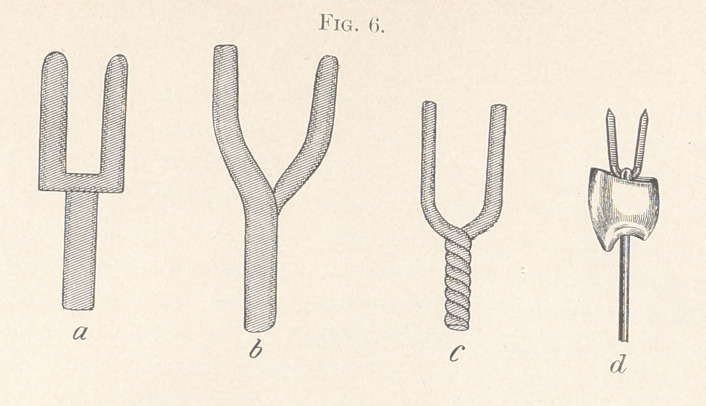


**Fig. 7. f7:**
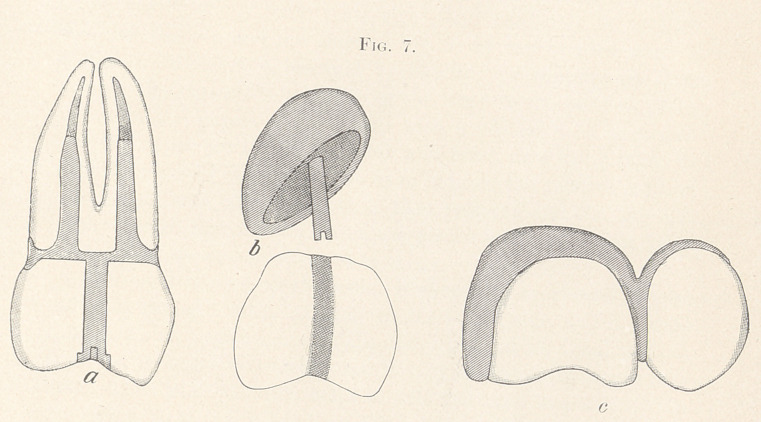


**Fig. 8. f8:**
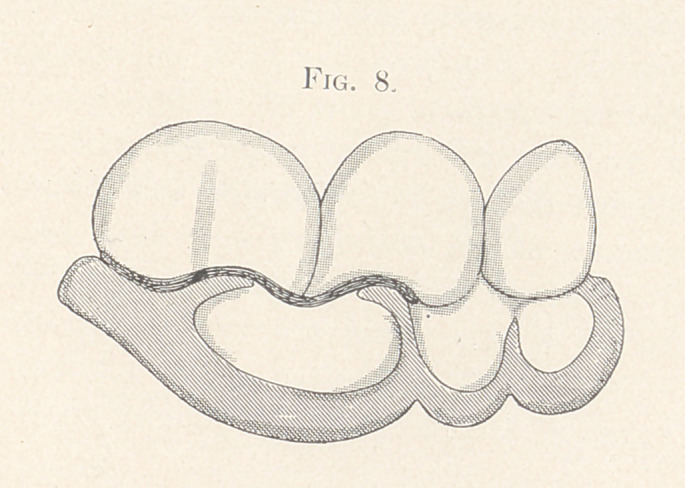


**Figure f9:**